# Sensory Changes Following Labor Epidural Analgesia: A Case Report

**DOI:** 10.1002/ccr3.72576

**Published:** 2026-04-19

**Authors:** Sara Gier, Jose L. Diz Ferre, Sabry Ayad

**Affiliations:** ^1^ Ohio University Heritage College of Osteopathic Medicine, Warrensville Heights Warrensville Heights Ohio USA; ^2^ Cleveland Clinic Fairview Hospital, Outcomes Research Consortium Cleveland Ohio USA

**Keywords:** anesthesia, epidural analgesia, obstetrics/gynecology, sensory changes

## Abstract

Understanding neurological adverse events is central not only for diagnosing and anticipating complications but also for informing patients accurately about the risks and benefits of epidural analgesia. Continued monitoring of neurological function with physical examination during and after the procedure is necessary.

## 1 Introduction

Epidural analgesia is an important means of pain control during labor, offering targeted pain relief that enhances patient satisfaction and overall childbirth experience. Although modern labor analgesia is not consistently associated with significant adverse outcomes for the parturient or fetus, it has been implicated in undesirable outcomes such as prolongation of labor and increased need for operative delivery [1]. The potential for rare neurological complications requires consideration. These complications can be the result of needle or catheter trauma, drug toxicity, spinal epidural hematoma, or infection, and may involve injury to the spinal cord, nerve roots, or neuraxial vasculature [2]. Other complications can include unintended intravascular or intrathecal injections and local anesthetic systemic toxicity. This case report examines the occurrence of transient bilateral lower facial and oral sensory changes during labor epidural analgesia, underlining the importance of vigilant monitoring to ensure patient safety.

## 2 Case Presentation

This report describes the case of a 31‐year‐old female, gravida 2, para 1, at 38 weeks' gestation, who requested epidural analgesia for labor. Her medical history was significant for two previous uneventful labor epidurals, chronic hypertension, asthma, and a smoking history of 11.5 pack/years before her pregnancy.

## 3 Methods

The patient was admitted to the birthing center for induction of labor (IOL) due to oligohydramnios. She was 2.5 cm dilated and 0.06 units/mL oxytocin infusion was started at 4 mL/h six hours before epidural, which was progressively increased. At the time of epidural, the patient was receiving the same concentration at 20 mL/h of oxytocin and tolerating it well. Vital signs at the time of epidural were blood pressure (BP) 139/76 mmHg, pulse 82, respiratory rate 16, and SpO2 98% at room air. It was performed in a sitting position using a non‐ultrasound‐guided, midline approach at the lumbar epidural interspace between the third and fourth vertebrae. Two attempts were required to place the 17G needle, which was inserted to a depth of 6 cm. An end‐hole 19G catheter was subsequently inserted to a skin depth of 13 cm without any complications. A test dose of 3 mL lidocaine 1.5% with epinephrine 1:200,000 was negative for intrathecal or intravascular injections. The loading dose included 16 mL bupivacaine 0.125% along with fentanyl 5 mcg/mL and epinephrine 1.25 mcg/mL in 10 mL NaCl 0.9%. No heme or cerebrospinal fluid (CSF) aspiration was noted. The patient tolerated the procedure well and did not experience any paresthesia during catheter insertion or test dose injection.

The epidural infusion of bupivacaine 0.0625% with fentanyl 2 mcg/mL ran for almost 6.5 h during the night, yielding a total of 58.97 mL. However, she reported new bilateral lower facial and mouth/tongue sensory changes in the first 2 h. At that time, another test dose of 3 mL lidocaine 1.5% with epinephrine 1:200,000 was negative. The baby's heart rate remained stable at 145 during this period. The patient had no previous history of similar symptoms. She described symptoms as pins and needles paresthesia without motor changes. There was no evidence of Horner syndrome or tinnitus [3]. She had full range of motion of her tongue, normal upper extremity strength, and normal sensation to touch and cold in her upper extremities and neck. Since the patient reported getting a little better and good pain relief, discontinuing the epidural was not necessary.

During IOL, oxytocin was paused secondary to tachysystole and late deceleration. They both resolved with administration of 250 mL lactated ringer bolus. The patient's vital signs were BP 139/76 mmHg, pulse 82, respiratory rate 16, and SpO2 98%, at room air. Fetal scalp electrode and intrauterine pressure catheter were placed, and oxytocin was restarted due to 109 Montevideo units per protocol until delivery. Once the cervix was found fully dilated, the infant and placenta were successfully delivered. The infusion was also stopped. After ~2 h, the epidural was removed, and the patient's vital signs were BP 136/85 mmHg, pulse 92, respiratory rate 19, and SpO2 98% at room air.

## 4 Conclusions and Results

The duration of the symptoms started 2 h after epidural infusion and slowly improved over time. After delivery and epidural removal, the patient was re‐evaluated. On repeat examination, she was neurologically intact, with normal cranial nerve II‐XII findings, equal and symmetric sensory findings, and full strength in both upper and lower extremities. The patient was reassured that her symptoms were not likely secondary to the neuraxial block. An episode of asthma was effectively treated with albuterol as needed during inpatient stay. She remained afebrile with stable vital signs throughout the postpartum period and was discharged safely.

## 5 Discussion

This case report details the experience of a 31‐year‐old female, who developed transient bilateral lower facial and oral sensory changes after 2 h of epidural analgesia during labor. These symptoms were characterized by tingling and numbness on the sides of her tongue. This raised the question of potential local anesthetic systemic toxicity (LAST) and other differential diagnoses [4].

Transient neurological symptoms (TNS) is a syndrome including pain and/or dysesthesia in the buttocks or lower extremities several hours after resolution of uncomplicated spinal anesthesia [5]. It is more common with lidocaine or mepivacaine compared to other local anesthetics [6]. For spinal anesthesia, a meta‐analysis reported greater risk of TNS with lidocaine compared to bupivacaine, prilocaine, or procaine, but similar risk with 2‐chloroprocaine and mepivacaine [7]. However, the occurrence of sensory changes in lower face and mouth/tongue did not align with this syndrome.

Bupivacaine's high lipid solubility and potency increase its affinity for neural tissues, and when absorbed systemically in excessive amounts, it can disrupt normal neural electrical activity [4]. The patient's symptoms gradually improved after discontinuation of the epidural, aligning with the resolution of mild systemic toxicity. The occurrence of mild sensory changes without severe systemic symptoms may have been consistent with early or low‐grade LAST, supporting bupivacaine as the possible cause given its continuous administration and pharmacological properties. We acknowledge the possibility of systemic anesthetic dissemination, since an unrecognized intrathecal administration could have happened considering that the epidural required several attempts. Bupivacaine has a narrow therapeutic index, which can increase the risk of LAST due to cumulative systemic absorption. Although she did not exhibit more severe CNS or cardiovascular symptoms such as seizures or hypotension, the patient's bilateral lower facial and tongue numbness could suggest mild systemic toxicity.

In contrast, lidocaine, used for the test dose with epinephrine, works similarly to bupivacaine by blocking sodium channels to inhibit nerve conduction. CNS toxicity from lidocaine typically manifests early due to the high sensitivity of neural tissues. Signs include circumoral and tongue numbness, metallic taste, tinnitus, dizziness, and visual disturbances [8]. Although the patient's tongue numbness could be an early indicator of lidocaine‐induced toxicity, the test dose of lidocaine was administered in a much smaller quantity. Additionally, the absence of more severe symptoms such as seizures or significant cardiovascular changes suggests that if lidocaine toxicity occurred, it was minimal and short‐lived [4]. However, the transient symptoms after 5 mL of lidocaine may also be the primary cause due to pregnancy susceptibility [9].

The preference for epidural analgesia in managing labor and cesarean section pain has increased due to its effectiveness. Despite this advantage, the technique carries inherent risks. Strategies to mitigate this risk include aspiration checks before administering each dose, fractionating doses, and the use of an epinephrine test dose [10]. Vigilant monitoring and prompt recognition of early possible signs of LAST complications during epidural analgesia are central. The transient bilateral lower facial and oral sensory changes observed in the patient would suggest mild systemic toxicity. Immediate attention to any neurological symptoms with appropriate follow‐up to reassure patients should not be underestimated. Epidural test dose and neurologic physical examination ensure safety during epidural blocks.

## Author Contributions

**Sara Gier:** conceptualization, writing – original draft. **Jose L. Diz Ferre:** conceptualization, writing – original draft. **Sabry Ayad:** conceptualization, writing – review and editing.

## Funding

The authors have nothing to report.

## Consent

Written informed consent was obtained from the patient for publication of the case along with accompanying images, according to the journal's policy.

## Data Availability

The data that support the findings of this study are available on request from the corresponding author. The data are not publicly available due to privacy or ethical restrictions.
